# A masked least-squares smoothing procedure for artifact reduction in scanning-EMG recordings

**DOI:** 10.1007/s11517-017-1773-0

**Published:** 2018-01-11

**Authors:** Íñigo Corera, Adrián Eciolaza, Oliver Rubio, Armando Malanda, Javier Rodríguez-Falces, Javier Navallas

**Affiliations:** Department of Electrical and Electronic Engineering, Public University of Navarra, 31006 Navarra, Spain

**Keywords:** Electromyography, Scanning-EMG, Signal processing, Motor unit

## Abstract

Scanning-EMG is an electrophysiological technique in which the electrical activity of the motor unit is recorded at multiple points along a corridor crossing the motor unit territory. Correct analysis of the scanning-EMG signal requires prior elimination of interference from nearby motor units. Although the traditional processing based on the median filtering is effective in removing such interference, it distorts the physiological waveform of the scanning-EMG signal. In this study, we describe a new scanning-EMG signal processing algorithm that preserves the physiological signal waveform while effectively removing interference from other motor units. To obtain a cleaned-up version of the scanning signal, the masked least-squares smoothing (MLSS) algorithm recalculates and replaces each sample value of the signal using a least-squares smoothing in the spatial dimension, taking into account the information of only those samples that are not contaminated with activity of other motor units. The performance of the new algorithm with simulated scanning-EMG signals is studied and compared with the performance of the median algorithm and tested with real scanning signals. Results show that the MLSS algorithm distorts the waveform of the scanning-EMG signal much less than the median algorithm (approximately 3.5 dB gain), being at the same time very effective at removing interference components.

**Graphical Abstract:**
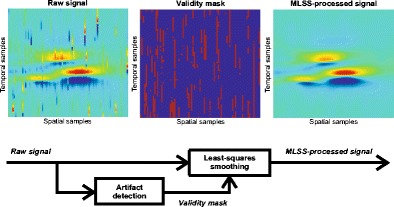
The raw scanning-EMG signal (left figure) is processed by the MLSS algorithm in order to remove the artifact interference. Firstly, artifacts are detected from the raw signal, obtaining a validity mask (central figure) that determines the samples that have been contaminated by artifacts. Secondly, a least-squares smoothing procedure in the spatial dimension is applied to the raw signal using the not contaminated samples according to the validity mask. The resulting MLSS-processed scanning-EMG signal (right figure) is clean of artifact interference.

The raw scanning-EMG signal (left figure) is processed by the MLSS algorithm in order to remove the artifact interference. Firstly, artifacts are detected from the raw signal, obtaining a validity mask (central figure) that determines the samples that have been contaminated by artifacts. Secondly, a least-squares smoothing procedure in the spatial dimension is applied to the raw signal using the not contaminated samples according to the validity mask. The resulting MLSS-processed scanning-EMG signal (right figure) is clean of artifact interference.

## Introduction

**Fig. 1:**
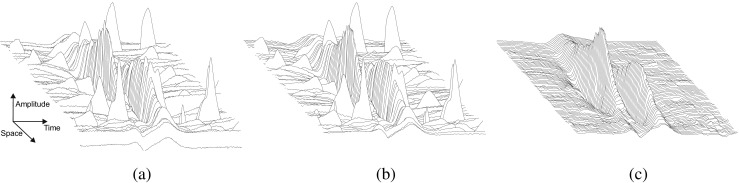
A three dimensional representation of a scanning-EMG signal for the different steps of the traditional signal processing procedure. **a** Raw scanning-EMG signal. **b** Scanning-EMG signal after applying a temporal band-pass filter. **c** Scanning-EMG signal after applying a 7-point spatial median filter, which is the output of the processing

Scanning-EMG is an electrophysiological technique that records the electrical activity of the motor unit (MU) in multiple spatial locations along a linear corridor [30]. A concentric needle, the scanning electrode, passes through the MU territory in small steps and records the motor unit potential (MUP) at each recording site. The technique uses a second needle that is placed a few centimeters away from the scanning electrode in the direction of the muscle fibers. This second needle, the trigger electrode, records the activity of the MU under analysis, and thereby makes it possible to synchronize a recording of the MUPs with the firing of the MU. The scanning-EMG signal can thus be viewed as a bi-dimensional MUP varying both in time and in space [20] (Fig. 1a).

Analysis of scanning-EMG signals has proved useful not only in the study of neuromuscular pathologies [3, 7, 8, 10, 12, 13, 33] but also, and specially, in the detailed characterization of the anatomical properties of the MU [3, 10, 17, 30, 32, 35]. Scanning-EMG signal analysis results in extraction from the signal of several useful descriptive parameters, such as the length of the MU territory [10], the number of MU fractions [31], the temporal delay between fractions [17], and the number of silent zones [31]. More recently, the motor unit profile has been proposed as another way to represent the scanning-EMG signal [2].

Reliable information, however, cannot be extracted from a raw scanning-EMG signal because such a signal is contaminated with noise and interference (Fig. 1a). There are three different types of noise in scanning-EMG recordings. First, there is baseline noise caused by needle or muscle movements [10, 11, 20]; second, there is high frequency noise due to the electronics of the acquisition system; and third, and most troublesome, there is the presence of artifacts deriving from the activation of nearby motor units during MUP recording at each position [10, 11, 20]. The level of interference from these other MUs depends on the level of voluntary contraction exerted by the subject or patient while the recording is being made [21].

Noise characteristics in biomedical signals, which will vary depending on the kind of signal and acquisition system employed, largely determine the suitable signal processing technique to be used [29]. For instance wavelet-based methods can be used when dealing with baseline noise [23] or speckle noise [27, 28], and median filter can be used to remove impulsive noise.

Noise and interference elimination has typically two steps in scanning-EMG recordings [10, 11, 20]. The first step is to apply a temporal band-pass filter to each of the scanning traces [10, 11, 20] (Fig. 1b). This filter removes the baseline noise in the low frequency range of the spectrum and some of the high-frequency noise from the acquisition system. The second step is to apply a median filter (usually of 3, 5, or 7 points) in the spatial dimension in order to eliminate the artifacts (Fig. 1c). Artifact interference falls within the frequency range of the physiological scanning-EMG signal in the temporal dimension, but it does not in the spatial dimension. This is because artifacts are not synchronized with the firing of the MU being tracked, and therefore, they are not consistently repeated in different traces, i.e., in the spatial dimension, they are effectively impulsive noise. This is the reason why the median filter applied in the spatial dimension is so effective at cleaning up scanning-EMG signals [11].

The median filter, however, has an important drawback: it considerably distorts the shape of the scanning-EMG signal. Peaks of the signal tend to be clipped significantly when the median filter is applied [10, 11, 20]. Peak clipping can be reduced by using a median filter with fewer points, but the fewer the points, the less effectively the artifacts are eliminated [10, 11]. Signal waveform distortion caused by the median filter can induce errors in subsequent analysis of the scanning-EMG signal.

In view of the above limitations inherent to the median filter, we suggest an alternative approach should be used for processing scanning-EMG signals. We propose a new processing algorithm—we will refer to it as masked least-squares smoothing (MLSS)—which recalculates and replaces each sample value of the scanning signal using a spatial least-squares smoothing procedure, taking into account information from only those samples that are not contaminated with artifacts. The algorithm is designed in such a way that it achieves a smooth waveform in the spatial dimension, exploiting the fact that in absence of noise and artifacts, the scanning-EMG signal is not expected to present abrupt variations, since the dependence between the recording position and the amplitude of the MUP is smooth [15]. In the present work, the MLSS algorithm is described, and its performance with both simulated and real scanning-EMG signals is analyzed and compared with that of the median algorithm.

## Materials and methods

### Algorithm description

**Fig. 2:**
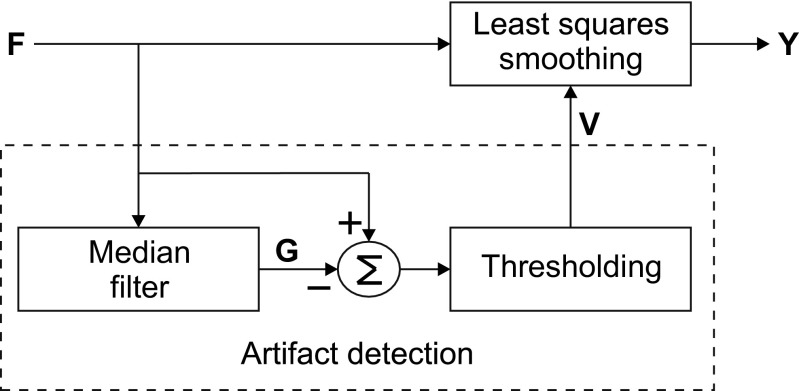
Block diagram describing the main steps of the MLSS algorithm. The input of the MLSS is the filtered signal ***F***, which is the result of applying the temporal filter to the raw signal. The filtered signal is used to generate the output signal ***Y*** by means of a local least-squares smoothing procedure in the spatial dimension. This procedure uses only the samples of the scanning-EMG signal that are considered clean, that is, to be free of artifacts as determined by a validity mask ***V***. To calculate ***V***, the algorithm prepares a signal ***G*** by twice applying, to the filtered signal ***F***, a median filter in the spatial dimension. Signal ***G*** is then subtracted from signal ***F***, and this difference, on the basis of a threshold, gives the validity mask ***V***

As is the case with median filtering in traditional procedure, the MLSS algorithm is used after applying a band-pass filter in the temporal dimension to reduce baseline and high-frequency noise. The MLSS algorithm has two steps: artifact detection and least-squares filtering (Fig. 2).

#### Artifact detection

The first step of the MLSS algorithm is to detect the samples in which the scanning-EMG signal is contaminated by artifacts (Fig. 2). Such interference is estimated by obtaining the difference between the *N* × *K* scanning-EMG signal before, ***F*** = {*f*_*n*,*k*_}, and after *L*-point spatial median filtering, ***G*** = {*g*_*n*,*k*_}, where *n* and *k* denote the temporal and spatial location of the sample, and *N* and *K* are the number of temporal and spatial samples of the recording.

This median filtering is applied twice consecutively in order to achieve more thorough artifact removal, that is

1 $$\begin{array}{@{}rcl@{}} {g}_{n,k}^{\prime} = \text{median} (f_{n,k-(L-1)/2}, {\ldots} , f_{n,k+(L-1)/2}) \end{array} $$

2 $$\begin{array}{@{}rcl@{}} g_{n,k} = \text{median} ({g}_{n,k-(L-1)/2}^{\prime}, {\ldots} ,{g}_{n,k+(L-1)/2}^{\prime}) \end{array} $$

The artifact estimation is then thresholded to obtain the *N* × *K* validity mask ***V*** = {*v*_*n*,*k*_}, which is set to 1 when an artifact has not been detected, and 0 otherwise

3 $$ v_{n,k}=\left\{\begin{array}{ll} 1, & |f_{n,k}-g_{n,k}|<U\cdot(\max (\boldsymbol{G}) - \min (\boldsymbol{G}))\\ 0, & |f_{n,k}-g_{n,k}|\ge U\cdot(\max (\boldsymbol{G})- \min (\boldsymbol{G})) \end{array}\right.  $$

where *U* is the normalized artifact detection threshold.

#### Spatial least-squares smoothing

The second step of the algorithm is to obtain the smoothed version of the scanning signal using only information from contamination-free samples (Fig. 2). For each sample at (*n*,*k*), a polynomial of order *Q* is fitted to the amplitude values of samples contained in a spatial window of length 2*M* + 1 centered at the spatial position under consideration. The value of the polynomial in the center of the window is taken as the filtered output at (*n*,*k*). Mathematically, the polynomial values within the window are

4 $$ p_{m} = \beta_{0} + \sum\limits_{q = 1}^{Q} \beta_{q} m^{q}, \,\,\,\, -M \leq m \leq M  $$

where *Q* is the polynomial order, *M* is the window semi-length, and ***β*** = [*β*_0_,…,*β*_*Q*_]^*T*^ are the coefficients of the polynomial.

When fitting the polynomial, therefore, the goal is to find the set of coefficients ***β*** that maximizes the fit of the polynomial to the values of the samples in the window. A weighted linear least-squares procedure [34] is used for this purpose. Samples marked as artifact contaminated are given weight 0, and contamination-free samples (marked as valid) are given weight 1. Thus, the mathematical expression to be minimized is [34]

5 $$ \underset{\boldsymbol{\beta}}{\arg\min}||\boldsymbol{W}^{1/2}(\boldsymbol{f-S\beta})||  $$

where ***f*** = [*f*_*n*,*k*−*M*_,…,*f*_*n*,*k*_,…,*f*_*n*,*k* + *M*_]^*T*^ are the window sample values, and the matrix ***S*** is defined as

6 $$ \boldsymbol{S} = \{s_{q,m}\}, \,\,\,\, s_{q,m}=\left\{\begin{array}{ll} m^{q} & q\ne 0\\ 1 & q = 0 \end{array}\right.  $$

with 0 ≤ *q* ≤ *Q* and − *M* ≤ *m* ≤ *M*. The weight matrix ***W*** is a diagonal matrix built from the validity mask values for samples within the window

7 $$ \boldsymbol{W} = \text{diag} \{v_{n,k-M}, {\ldots} ,v_{n,k}, {\ldots} ,v_{n,k+M}\}  $$

Polynomial coefficients ***β*** satisfying (5) can be calculated as [34]

8 $$ \boldsymbol{\beta} = (\boldsymbol{S}^{T} \boldsymbol{WS})^{-1} \boldsymbol{S}^{T}\boldsymbol{Wf}  $$

The overall *N* × *K* output signal ***Y*** = {*y*_*n*,*k*_} comprises the series of values of the polynomials at the center of the windows (i.e., in *m* = 0). Therefore, using (4) we obtain

9 $$ y_{n,k}=p_{0}=\beta_{0}  $$

For spatial positions where there are not *M* traces on each side, and therefore it is not possible to set the sample window symmetrically, output signal values are taken from the polynomial obtained for the nearest entire window which includes the bordering spatial position. Thus, for 1 ≤ *k* ≤ *M*, the output values are

10 $$ y_{n,k}=p_{k-M-1}  $$

where the polynomial values are obtained from the sample window centered at the position (*n*,*M* + 1). Similarly, for *K* − *M* + 1 ≤ *k* ≤ *K*, the output values are

11 $$ y_{n,k}=p_{k-K+M + 1}  $$

where the polynomial values are obtained from the sample window centered at the position (*n*,*K* − *M*).

Another consideration in least-squares fitting is that, in order to obtain a unique solution, the chosen polynomial order must be smaller than the number of samples used [34]. In the algorithm proposed here, a more restrictive condition is imposed so as both to ensure the uniqueness of the solution, and to avoid incorrect polynomial solutions that can result from the use of a polynomial order that is high relative to the number of samples. The condition applied is

12 $$ Q<\frac{1}{2} \sum\limits_{m=-M}^{M}v_{n,k+m}  $$

For space-time positions in which this condition is not satisfied, the highest polynomial order *Q* that satisfies the condition is chosen.

### Algorithm evaluation

#### Model of scanning-EMG signals

Simulated scanning-EMG signals were used to evaluate the performance of the MLSS algorithm. In this section, the simulation model of scanning-EMG signals is described.

##### Muscle and motor unit modeling

Each muscle was created with a circular muscle cross section of 10 mm diameter and was composed of 120 MUs. The cross-sectional areas of MU territories were modeled fitting an exponential function [6] between the area of the smallest MU, 1.96 mm^2^, and the area of the largest MU, 22.48 mm^2^. MU territories were circular in shape except when constrained by the muscle boundary, in which case the MU territory was cut to fit within the muscle limits, and the radius was regrown so as to keep the territory area unchanged [14, 18, 19]. MU territories were placed within the muscle in such a way that the spatial variance of the overlapping between MU territories within the muscle was minimized [19, 26]. For each MU, muscle fibers were modeled according to a uniform distribution inside the MU territory [5], with a fiber density of 10 fibers/mm^2^. The length of muscle fibers was 140 mm. The conduction velocity of muscle fibers within the *i*^*th*^ MU were given by a Gaussian distribution, with a mean conduction velocity modeled by an exponential function [6] between the MU conduction velocity of the smallest MU, 3.25 m/s, and the MU conduction velocity of the largest MU, 6.25 m/s, and with a coefficient of variation 0.03.

**Fig. 3:**
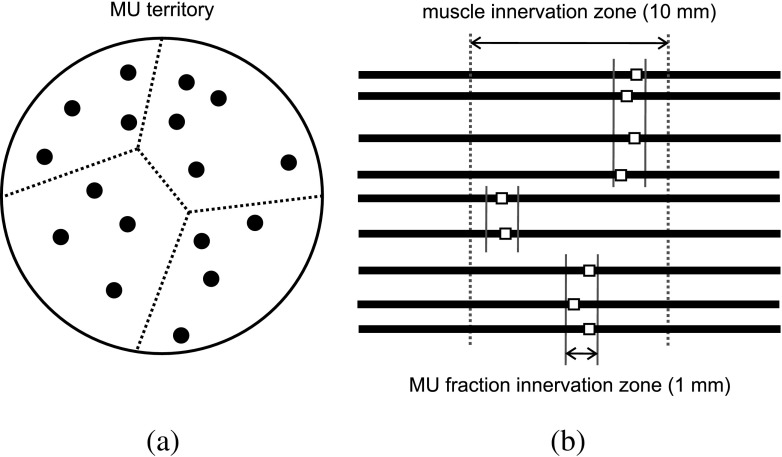
**a** Schematic representation of a simulated MU cross section. The MU is divided in different Vornoi cells (dotted lines), which correspond to the different fractions of the MU. The fibers of the MU are represented with filled black circles. **b** Schematic representation of the innervation zone of a MU of the simulated muscle. The horizontal black lines represent the different fibers of the MU. The innervation position of the fibers (represented by squares) follow a uniform distribution within the limits of each fraction zone (vertical solid lines). The different MU fraction zones follow a uniform distribution within the muscle innervation zone (vertical dashed lines)

The cross section of each muscle was divided into regions called fractions (Fig. 3). Given a specific motor unit, each fraction located within the MU territory represents the set of muscle fibers innervated by a common axonal branch [3, 17, 31]. A set of 90 points were created within the muscle cross section at random positions following a uniform distribution. From these points, Voronoi tessellation of the MU territories was performed [22], each Voronoi cell corresponding to a different fraction (Fig. 3a). Muscle fibers were assigned to fractions on the basis of position. The innervation zone center of the muscle was located in the middle, lengthwise, of the muscle fibers. Innervation zone centers for the different fractions within each MU were positioned according to a uniform distribution scattered 10 mm around the muscle innervation zone center (Fig. 3b). Innervation positions for muscle fibers followed a uniform distribution of 1 mm around the innervation zone center of the fraction of the MU in which the muscle fiber was located (Fig. 3b).

##### Recruitment and firing pattern modeling

The recruitment and firing pattern of each motor unit were modeled according to [5, 6]. The recruitment threshold of the *i*^*th*^ MU during constant isometric contraction was modeled by an exponential function [5, 6], with a percentage of voluntary contraction (MVC) of full recruitment of 70% [6]. The minimum firing rate was 8 pps (pulses per second), and the firing rate increased linearly with increased strength of voluntary contraction: at a rate of 7 pps for each 10% increase in MVC [5]. The maximum firing rate was 35 pps [5]. The firing train of each MU was modeled as a renewal point process where the interval between discharges followed a Gaussian distribution with a mean that was the inverse of the firing rate, and with a coefficient of variation of 0.15.

##### Scanning-EMG signal modeling

Single-fiber action potentials (SFAPs) were simulated using a line source model [24], and MUPs were generated by the summation of the individual SFAPs [16]. In order to simulate the effect of concentric needle recording [16], MUPs were calculated and averaged for a grid of points over the uptake area [16]. The cannula effect of the concentric needle was also taken into account by simulating and averaging the MUP in the cannula section; the resulting signal was subtracted from the core MUP [16].

The physiological scanning-EMG signal was simulated as the sequence of MUPs obtained at each position of the scanning corridor. The scanning-EMG signal was simulated from the smallest MU whose territory was traversed by the corridor. The modeled scanning electrode was inserted 30 mm away from the innervation zone. The scanning corridor was located across the plane of muscle cross section, from the bone to the skin, recording a signal every 0.05 mm [30].

In order obtain modeled scanning-EMG signals that were realistically noisy, the entire recording procedure was modeled. For each recording position, the EMG signal was calculated as the convolution of the MUPs of all recruited MUs and their corresponding firing trains. Each recording trace was a 30-ms duration segment of the complete EMG signal around the corresponding MU firing time. After each trace, a waiting time of 60 ms was emulated in order to model the time a scanning electrode takes to advance to the next recording position.

Baseline noise was modeled as an ARMA process [23] obtained by filtering white Gaussian noise of zero-mean. The filter used was a 5^*th*^-order Butterworth low-pass filter with a 3 dB cut-off frequency of 20 Hz. Electronic acquisition noise was simulated as a zero-mean, additive, white Gaussian noise process.

#### Influence of the algorithm parameters

The parameters of the MLSS algorithm are the artifact detection threshold, *U*, the median filter order, *L*, the polynomial order, *Q*, and the window semi-length, *M*. The first goal of the study was to find a set of optimal-performance parameters by using a genetic algorithm [9]. The cost function to be minimized was the error power within the physiological activity recording region, *P*_*in*_, after processing with the MLSS algorithm, averaged for 100 simulated scanning-EMG signals, where

13 $$ P_{in}= 10 \log_{10} \frac{{\sum}_{k = 1}^{K} {\sum}_{n = 1}^{N} z_{n,k}|e_{n,k}|^{2}}{{\sum}_{k = 1}^{K} {\sum}_{n = 1}^{N}z_{n,k}}  $$

where *e*_*n*,*k*_ is the error between the processed scanning-EMG signal and the physiological (noise-free) scanning-EMG signal. The mask *z*_*n*,*k*_ discriminates between what is inside and what is outside the region of physiological activity. For positions considered inside the physiological activity region, the mask is set to 1; otherwise, the mask is set to 0. For each trace at each spatial position, the region of physiological activity is defined as the interval between the first and last sample for which the signal exceeds 9% of the maximum amplitude value of the entire scanning-EMG signal:

14 $$ z_{n,k}=\left\{\begin{array}{ll} 1 & n_{a_{k}}\leq n\leq n_{b_{k}}\\ 0 & \text{otherwise} \end{array}\right.  $$

where $n_{a_{k}}$ and $n_{b_{k}}$ are for each *k*, the first and last samples that satisfy |*x*_*n*,*k*_| > 0.09 max(***X***). The percentage of MVC used in this experiment was 2.3%. The genetic algorithm was used with the following settings: the number of generations was 100, the population size was 50; the number of individuals belonging to the elite was 2; and the crossover probability was 0.8. The parameter search ranges can be found in Table 1 (Min. and Max. values).

**Table 1:** Ranges and values for the MLSS algorithm parameters

Parameter	Symbol	Min.	Max.	Optimal
Median filter order	*L*	3	11	5
Artifact detection threshold	*U*	8 ⋅ 10^− 3^	5.23 ⋅ 10^− 2^	2.23 ⋅ 10^− 3^
Polynomial order	*Q*	2	12	8
Window semi-length	*M*	7	18	13

In order to analyze the effect of the parameters in the algorithm performance, Sobol sensitivity analysis was performed [25]. Using Sobol analysis, the total variance of the average *P*_*in*_ of 100 scanning signals was decomposed in terms which can be attributed to each of the algorithm parameters or to combinations of them [25]. All order indices, including the total-effect indices, were calculated [1]. A total of 4000 samples of the parameter space were used to compute the indices. In this way, uniform random sampling of the parameter space was used, within the ranges of the algorithm parameters given in Table 1 (Min. and Max. values).

#### Influence of level of muscle contraction

In this section of the study, we compared the performance of median and MLSS algorithms over a range of levels of muscle contraction, the higher the muscle contraction, the higher the level of artifact contamination. In 100 independent realizations of the muscle model, a physiological scanning-EMG signal and a noisy scanning-EMG signal were simulated for a series of different percentages of MVC (from 1.4 to 4.2% in steps of 0.2%). The noisy signals were processed by the median and the MLSS algorithms. The median algorithm was used with three different median filter-orders (3, 5, and 7). The MLSS was used with the optimal parameters (Table 1) obtained from the experiment described in Section 2.2.2. Once the scanning-EMG signals had been processed, the following merit figures were calculated, in addition to *P*_*in*_:

- Error power outside the recording region where the physiological activity is located
  15 $$ P_{out} = 10\,\log_{10} \frac{{\sum}_{k = 1}^{K} {\sum}_{n = 1}^{N} (1-z_{n,k})|e_{n,k}|^{2}}{{\sum}_{k = 1}^{K} {\sum}_{n = 1}^{N}(1-z_{n,k})}  $$
- The difference between the 3-, 5-, and 7-point median algorithms and the MLSS algorithm in terms of the error power outside the physiological activity region, *G*_*out*_.
- The difference between the 3-, 5-, and 7-point median algorithms and the MLSS algorithm in terms of the error power within the physiological activity region, *G*_*in*_.

#### Error distribution in the recording region

The aim of this experiment was to analyze whether the error associated with an algorithm is uniformly distributed throughout the recording or whether such an error tends to be concentrated in certain regions. To this end, for both median and MLSS algorithms, we studied the statistical behavior of the remaining error after the processing at each sample. Only one simulated MU was used, but the scanning recording procedure with that MU was simulated in 1000 independent runs. The percentage of MVC was 2.3% and was not varied. The noisy scanning-EMG signal obtained in each run was processed by both algorithms and the error at the output of the algorithms *e*_*n*,*k*_ was calculated. To statistically characterize the error distribution in the recording region, we calculated the bias $\hat {\mu }_{n,k}$ and the standard deviation (SD) $\hat {\sigma }_{n,k}$ of the 1000 runs.

#### Application with real scanning-EMG signals

The applicability of the algorithm was tested using real scanning-EMG recordings. Note that, when dealing with real scanning-EMG signals it is not possible to objectively quantify the algorithm performance, as the physiological noise-free version of the recorded scanning-EMG signal is not available. This issue has already been discussed in other EMG studies [4]. It is possible, instead, to analyze the effects of the processing algorithms by comparing the waveform of the resultant scanning-EMG signals. To this end, four real cases are presented in this paper, in which a real scanning-EMG signal was processed by both the MLSS and the 7-point median algorithms. The MLSS algorithm was used with the optimal parameters shown in Table 1.

The real scanning-EMG signals used were selected from a set of 20 recordings performed in the biceps brachii of five normal subjects (3 male and 2 female) aged between 24 and 54 years. Informed consent was obtained from all subjects, and the study was approved by the ethics committee of Public University of Navarra. The scanning-EMG recordings were obtained following the recording protocol described in [30], with sampling frequency of 20 kHz and scanning steps of 50 *μ* m. A concentric needle was used as scanning electrode and a facial concentric needle was used as trigger electrode. The scanning electrode was inserted 30 mm away from the trigger electrode along the longitudinal axis of the muscle.

## Results

### Influence of the algorithm parameters

**Fig. 4:**
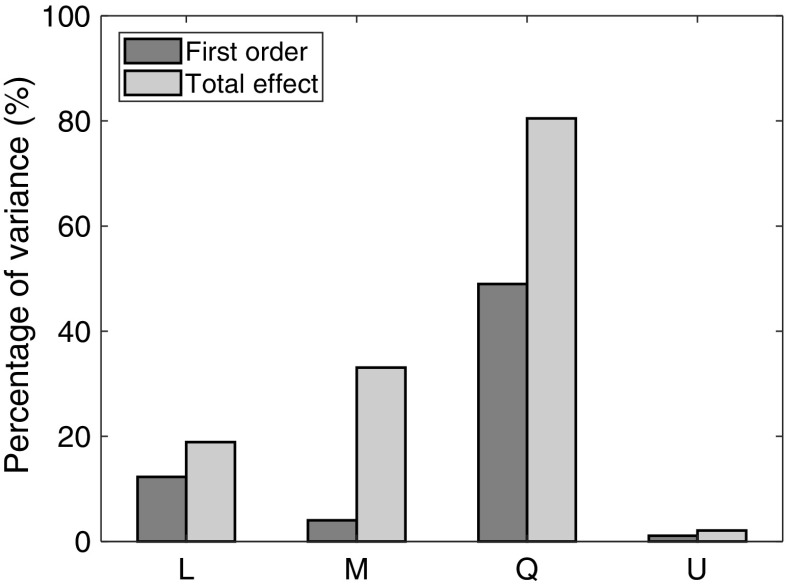
Results of the Sobol indices obtained in the sensitivity analysis. The first-order indices of all parameters are represented in dark gray, and the total-effect indices of the parameters are represented in light gray. The parameters of the MLSS algorithm evaluated are the artifact detection threshold, *U*, the median filter order, *L*, the polynomial order, *Q*, and the window semi-length, *M*

The optimal parameters obtained by means the genetic algorithm are: *L* = 5, *U* = 2.23 ⋅ 10^− 2^, *Q* = 8 and *W* = 13. In Fig. 4, the results of the Sobol sensitivity analysis about the first-order and total-effect indices of the parameters are depicted. The polynomial order *Q* has the highest sensitivity with the first-order index being 49% and the total-effect index 80.5% of the total variance. The effect of the window semi-length *M* and the median filter order *L* are also significant, with the total-effect indices being 33.1 and 18.9% respectively. The least-sensitive parameter is the artifact detection threshold *U*, whose total-effect index is 2.1%. With regard to higher order indices, only two of them are higher than 2% of the total variance: the interaction between *L* and *Q*, whose second-order index is 4.9%, and the interaction between *M* and *Q*, whose second-order index is 27.8%.

### Influence of level of muscle contraction

**Fig. 5:**
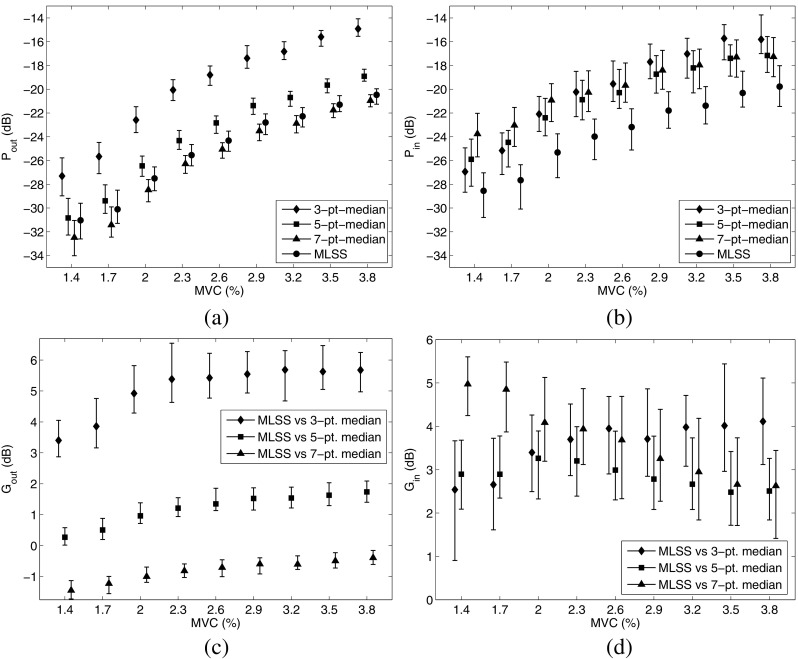
Boxplot representation of the merit figures obtained for different percentages of the MVC level. In the upper sub-figures the error powers *P*_*out*_ (**a**), and *P*_*in*_ (**b**) are represented for the median and the MLSS algorithms. In the lower sub-figures the differences *G*_*out*_ (**a**) and *G*_*in*_ (**b**) of the MLSS algorithm respect to the median one is represented

The results for error power outside (*P*_*out*_) and inside (*P*_*in*_) the physiologically active region at different levels of muscle contraction are represented in Fig. 5a, b. Note that the higher the percentage of the MVC level, the higher the error power for both algorithms. Regarding the median algorithm, the higher the median filter order, the lower the error power outside the physiologically active region. For the MLSS algorithm, the error power outside the physiologically active region was lower than that of the 3- and 5-point median algorithm and higher, although very close, to that of the 7-point median one. Within the physiologically active region, in contrast, the error power was lower than that of the median algorithm at all levels of contraction studied and for all orders of the median filter used in the median algorithm.

The results of *G*_*out*_ and *G*_*in*_ at different levels of muscle contraction are depicted in Fig. 5c, d. The lower the order of the median algorithm, the higher the *G*_*out*_, which was positive in the vast majority of runs with the 3- or 5-point median algorithm. The *G*_*out*_ median was ranged between 3.4 and 5.68 dB for the 3-point median algorithm, and between 0.27 and 1.74 dB for the 5-point median algorithm. With the 7-point median algorithm, *G*_*out*_ was negative for all MVC values but low in magnitude, ranging between − 1.44 and − 0.39 dB. Regarding *G*_*in*_, in the vast majority of the simulation runs, it was positive at all of the contraction levels investigated, and for any order of the median algorithm. The *G*_*in*_ median ranged between 2.55 and 4.11 dB with the 3-point median algorithm, between 2.48 and 3.26 dB with the 5-point median algorithm, and between 2.63 and 4.97 dB with the 7-point median algorithm.

### Error distribution in the recording region

**Fig. 6:**
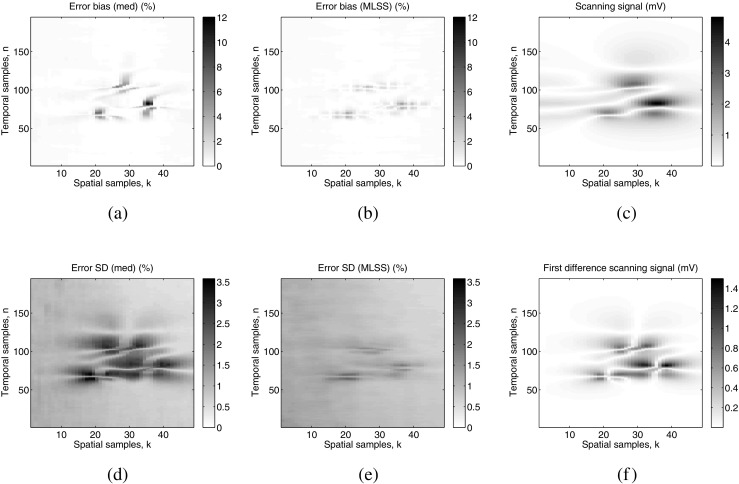
Results of the spatio-temporal distribution of the algorithm error for the scanning-EMG signal of the experiment described in Section 2.2.4 (a single fixed MU). **a** Error bias for the median algorithm in absolute value. **b** Error bias for the MLSS algorithm in absolute value. **c** Noise-free scanning-EMG signal in absolute value. **d** Error SD for the median algorithm. **e** Error SD for the MLSS algorithm. **f** First difference of noise-free scanning-EMG signal over the length of the spatial dimension, in absolute value. Note the relation between the statistical behavior of the error at the different spatio-temporal positions and the location of physiological activity in the recording region (**c**). Note that the error bias and the error SD are expressed in *%* of the amplitude of the scanning-EMG signal

The results of the spatio-temporal distribution of the bias and SD error for the experiment described in Section 2.2.4 (a single fixed MU) are shown in Fig. 6. The error bias (Fig. 6a, b) of both algorithms only differed significantly from zero in recording regions where there was physiological activity (Fig. 6c). The position of the most negative value of the error bias for the median algorithm coincides with the position of the highest peak of the scanning-EMG signal, which is located at *n* = 85, *k* = 35 (Fig. 6c). This means that the highest peak of the signal was systematically clipped by the median algorithm.

Regarding error SD (Fig. 6d, e), outside the region of physiological activity, this SD was approximately constant for both algorithms (between about 0.8 and 1.3%). Within the physiologically active region, however, the SD with the median algorithm (which reached 3.6%) was greater than outside this region (Fig. 6d). In fact, the distribution of the error SD for the median algorithm was strongly correlated with the first difference of the scanning-EMG signal in the spatial dimension (Fig. 6f), which means that regions of higher slope in the spatial dimension present a higher SD error. With regard the MLSS algorithm, on the other hand, the error SD within the physiological region barely increases respect to that of the non-physiological recording region (Fig. 6e).

### Application with real scanning-EMG signals

**Fig. 7:**
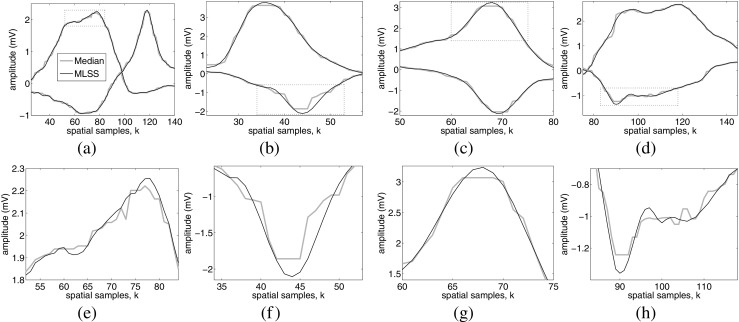
The amplitude profile in the spatial dimension of 4 real scanning-EMG recordings is represented for both the 7-point median (gray lines) and the MLSS algorithm (black lines). The upper sub-figures show the amplitude profile of the scanning-EMG signals and the lower ones the corresponding detail of the profile within the dotted lines rectangles. The amplitude profile of each signal is corresponded with the spatial traces in the time instants of maximum and minimum amplitude, respectively

**Fig. 8:**
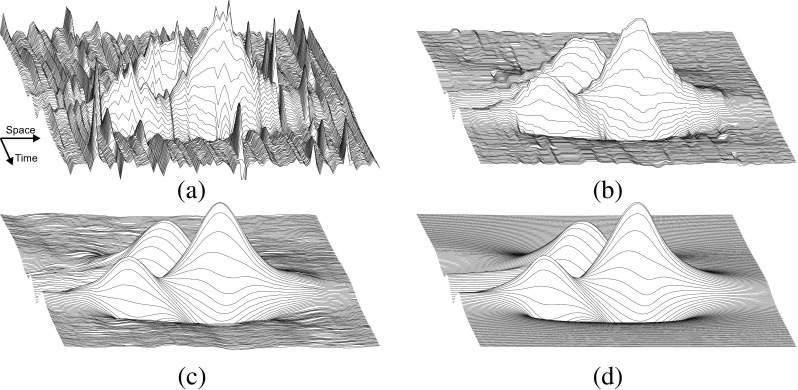
Simulated scanning-EMG signal processed using both algorithms: **a** raw signal; **b** after applying the 7-point median processing algorithm. *P*_*in*_ = − 52.3 dBm; **c** after applying the MLSS processing algorithm, *P*_*in*_ = − 55.4 dBm; **d** ideal noise-free scanning-EMG signal. Note that the lines of the graphs link spatial samples and not temporal samples, and thus they cannot be interpreted as MUPs

The output from the 7-point median algorithm and the MLSS algorithm after processing four real scanning-EMG signals is depicted in Fig. 7, where amplitude profiles are presented together with details of the profiles. In all cases the MLSS algorithm provides a smoother waveform than the median algorithm does. In fact, the median-filtered output is spiky and rather stepped in the spatial dimension (Fig. 7b, e, f, h). Peak clipping produced by the median algorithm is evident in Fig. 7b, f–h. In Fig. 8, a simulated scanning-EMG signal processed by both algorithms is shown, as an example. The simulated signal also presents a steeped amplitude profile in the spatial dimension when it is processed with the median algorithm.

## Discussion

### Algorithm performance

Potentials from nearby motor units are the principal source of contamination in scanning-EMG recordings [20], and a signal processing technique that effectively removes such contamination with low distortion is therefore required [10, 11, 20] so that subsequent analysis of the scanning-EMG signal is reliable and accurate. To this end, the current study presents and evaluates the MLSS algorithm compared to the median algorithm. Test results indicate that the algorithm removes artifacts from nearby motor units effectively and does so with low distortion of the scanning signal waveform, which is a major advantage over the median processing algorithm.

With regard to removal of artifact contamination, when dealing with the parts of a signal recorded outside the region of physiological activity, the error power of the MLSS is lower than that of the 3- or 5-point median algorithm. The error power of the MLSS is higher only when compared to the 7-point median algorithm, which we found to be the most effective of the median algorithms at contamination removal (Fig. 5a and c), these differences are however small (median *G*_*in*_ between − 1.44 and − 0.39 dB) (Fig. 5a, c).

Within the region of physiological activity, the error power of the MLSS algorithm is lower than that of the median algorithm for any order of the median filter in median algorithm (Fig. 5b, d); the median of *G*_*in*_ can be up to 4.9 dB when comparing to the 7-point median algorithm (Fig. 5d). This suggests that the MLSS algorithm distorts the physiological waveform less than the median algorithm does. With regard to distortion, the MLSS algorithm performed better than the median algorithm over a wide set of scanning signals and at increasing amounts of artifact contamination resulting from higher levels of simulated muscle contraction (Fig. 5b, d).

It is well known that the distortion produced by the median filter tends to clip the peaks of a scanning-EMG signal [10, 11, 20]. In the trials with real scanning-EMG signals, peak clipping was observed for the median algorithm but not for the MLSS algorithm (Fig. 7). In the tests with simulated signals (Section 3.3), the error distribution of the median algorithm had negative bias in regions where there were scanning-EMG signals peaks (Fig. 6a, c in *n* = 85, *k* = 35), which implies that peak clipping was systematically occurring in these zones throughout the multiple runs of the simulation experiment. In the case of the MLSS algorithm (Fig. 6b), the corresponding bias values were lower than those of the median algorithm (bias between − 12% and 5.95% of maximum amplitude for the median, and between − 4.83% and 3.9% for the MLSS), indicating that distortion was less severe (Fig. 6a).

Another kind of distortion in scanning-EMG signals processed with the median algorithm was stepping in the amplitude profile of the scanning signal in the spatial dimension. To the best of our knowledge, this effect has not been described before in published studies; we observed the effect in both simulated (Fig. 8b) and real scanning signals (Fig. 7b, e, f, h). Stepping was associated with an increase in error SD in the recording area where physiological activity was located, especially in regions of the scanning signal with a pronounced slope in the spatial dimension (Fig. 6d, f). This is consistent with the fact that the 7-point median algorithm presents a significantly higher error power within the physiologically active region than outside of it (Fig. 5a and b). With regard to the MLSS algorithm, the processed signals presented a smooth waveform (Fig. 7) which, in the case of simulated signals (Fig. 8c), was similar to that of the noise-free ideal scanning-EMG signal (Fig. 8d, *P*_*in*_ = − 55.4 dBm). Accordingly, inside the physiologically active region, error SD with the MLSS algorithm was low, in fact, the difference between the error SD inside and outside the physiologically active region is small as can be observed in Fig. 6e.

### Parameter settings

With regard to selecting a suitable set of parameters, the sensitivity study indicates that the algorithm performance is very influenced by the polynomial order *Q* (being its first-order index 49% of the total variance), and also by the interaction between the window semi-length *M* and *Q* (27.8%). The tradeoff between *Q* and *M* determines the ability of the polynomial to track variations of the scanning-EMG signal in the spatial dimension. The higher the *Q*, or the lower the *M*, the greater the ability to track fast variations, which implies less waveform distortion when dealing with sharp scanning signals, but also less ability to remove artifact contamination. The median filter order, *L* has significant influence in the algorithm performance (first-order index 12.3%), but the artifact detection threshold *U* it is not, at least in the range of parameters studied. These two parameters are related with the artifact detection effectiveness. A high *L* value involves a better artifact elimination, but it may also cause waveform distortion in sharply scanning signals, due to false artifact detections in the signal peaks.

Thus, the suitable parameter configuration will depend on the recording conditions of the scanning-EMG signal. For instance, when dealing with scanning signals presenting sharp peaks in the spatial dimension, parameter settings that imply low distortion should be selected. On the other hand, if the recorded signal has a very high level of artifact contamination, it may be desirable to select a parameter configuration that prioritizes the noise elimination instead. Despite that, the optimal algorithm parameters obtained in our study (see Table 1) can in the first instance be a good configuration to be routinely used, as using these parameters, the algorithm was able to work properly in scenarios with different levels of artifact contamination (different levels of voluntary contraction) and with a large set of scanning-EMG signals.

## Conclusion

An algorithm based on masked least-squares smoothing has been proposed for and evaluated in the processing of scanning-EMG signals. Simulation experiments show that the new algorithm overcomes limitations of the median algorithm: stepping in the amplitude profile and peak clipping. Furthermore, the tests indicate that, over the studied range of muscle contraction levels, the new algorithm performed with noticeably less distortion of the signal waveform than the median algorithm, while effectively removing noise and artifacts from nearby MUs.
